# The role of vitamin D in increasing circulating T regulatory cell numbers and modulating T regulatory cell phenotypes in patients with inflammatory disease or in healthy volunteers: A systematic review

**DOI:** 10.1371/journal.pone.0222313

**Published:** 2019-09-24

**Authors:** Sheila A. Fisher, Mana Rahimzadeh, Charlotte Brierley, Betty Gration, Carolyn Doree, Catherine E. Kimber, Alicia Plaza Cajide, Abigail A. Lamikanra, David J. Roberts

**Affiliations:** 1 NHS Blood and Transplant, Oxford, United Kingdom; 2 Nuffield Division of Clinical Laboratory Sciences, Radcliffe Department of Medicine, University of Oxford, Oxford, United Kingdom; 3 Oxford University Medical School, John Radcliffe Hospital, Oxford, United Kingdom; 4 Department of Haematology, Oxford University Hospitals NHS Foundation Trust, Churchill Hospital, Oxford, United Kingdom; 5 Department of Haematology, Guy's and St Thomas' NHS Foundation Trust, London, United Kingdom; 6 Biomedical Research Centre (Haematology Theme), Oxford University Hospitals NHS Trust, Churchill Hospital, Oxford, United Kingdom; Telethon Kids Institute, AUSTRALIA

## Abstract

**Background:**

The evidence for vitamin D and other agents that experimentally modulate T regulatory cells (Tregs) for the treatment of patients with autoimmune or allergic diseases has not been established.

**Objective:**

We have undertaken a systematic review of randomised controlled trials to assess the efficacy of vitamin D, vitamin A, niacin and short-chain fatty acids in enhancing absolute Treg numbers and phenotypes in patients with inflammatory or autoimmune disease.

**Methods:**

This systematic review was conducted using a predefined protocol (PROSPERO International prospective register of systematic reviews, ID = CRD42016048648/ CRD42016048646). Randomised controlled trials of patients with inflammatory or autoimmune disease or healthy participants which compared either oral vitamin D or vitamin A or short-chain fatty acids with control or placebo and measured the absolute concentration of proportion of Tregs were eligible for inclusion. Searches of electronic databases (CENTRAL, MEDLINE, EMBASE, CINAHL, PUBMED and Web of Science) identified eight eligible independent trials (seven autoimmune disease trials, one trial of healthy subjects). Data were extracted by two reviewers and the risk of study bias was assessed using Cochrane Collaboration methodology.

**Results:**

Planned meta-analysis was not possible due to the heterogeneous nature of the studies. Nevertheless, in five trials of autoimmune disorders which measured the proportion of Tregs, a higher proportion was observed in the vitamin D group compared to controls at 12 months in all but one trial. In the trial of healthy subjects, a significant difference was reported, with a higher percentage of Tregs observed in the vitamin D group (at 12 weeks, mean 6.4% (SD 0.8%) (vitamin D) vs 5.5% (1.0%) (placebo). There were no trials to assess the efficacy of vitamin A, niacin and short-chain fatty acids in enhancing absolute Treg numbers.

**Conclusions:**

Vitamin D supplementation may increase Treg/CD3 ratios in both healthy individuals and patients with autoimmune disorders and may increase Treg function. There remains a need for further suitably powered clinical studies aimed at enhancing Treg numbers and/or function.

## Introduction

Vitamin D exerts profound effects beyond its classical actions on bone metabolism [1, 2]. There is now increasing interest in repurposing vitamin D to down-regulate pathological immune responses in patients in autoimmune or inflammatory disease. Higher levels of vitamin D may induce many different anti-inflammatory functions including increasing the number and/or function of T regulatory cells (Tregs). Moreover, experimental studies have suggested other small molecules including vitamin A, niacin and short-chain fatty acids may enhance Tregs. However, the relationship between vitamin D therapy or other small molecules and changes in Treg numbers or function in patients or healthy volunteers has not well-defined.

Vitamin D is a prohormone that comes in two forms: vitamin D2 (VD2, ergocalciferol) found mostly in plants and vitamin D3 (VD3, cholecalciferol) found in animal-based food. VD3 is synthesised in human epidermis by ultraviolet B conversion of 7-dehydrocholesterol (provitamin D3) to previtamin D3 followed by thermal isomerisation to generate VD3 (for reviews see [3] and [4]). Both VD2 and VD3 are hydroxylated in the liver to form 25(OH) vitamin D [25(OH)D] by CYP2R1 (25-hydroyxlase) and then further hydroxylated by a renal 1-alpha-hydroxylase to produce the biologically active hormone 1,25(OH)2 vitamin D [1,25(OH)2D]. The 25(OH)D metabolite is the major circulating form of vitamin D and is bound to vitamin D binding protein with a half-life of 15 days compared to that of 1,25(OH)2D which is measured in hours. So, the concentration of plasma 25(OH)D is considered the best indicator for the bioavailability of vitamin D and the vitamin D status of individuals [5]. Currently vitamin D deficiency is defined as < 50nM 25(OH)D [6].

Cholecalciferol has profound effects on a wide variety of cellular functions [1]. Historically, attention has been focused on the role of the active metabolite in bone metabolism where 1,25(OH)2D can stimulate osteoblasts, increase calcium ion absorption from the renal tubule and gut and so stimulate the formation of new cancellous bone [7]. The presence of the vitamin D receptor (VDR) in activated T cells first suggested that vitamin D may have a role in the function of the immune system [8]. Indeed, vitamin D receptors are present in a wide variety of cell types, stimulating interest in the role of 1,25(OH)2D not only in immune function but also host defence, cardiovascular disease and cancer [1, 9, 10]. More recently, genomic studies have shown that 1,25(OH)2D can up- or down- regulate hundreds of genes through binding of 1,25(OH)2D and VDR to regulatory elements to recruit co-activators or co-repressors [11]. However, the clinical evidence for using formulations of VD2 and VD3 to modulate immune cell function, the inflammatory response or treatment of diseases due to dysregulation of the immune system is limited.

Vitamin D3 may induce tolerogenic dendritic cells that may in turn stimulate IL-10 producing CD4+ T-cells and antigen-specific Tregs [12]. High levels of 1,25(OH)2D can induce the lineage-specific FOXP3 transcription factor involved in the development and function of Tregs [13, 14] and so enhance the number and/or function of circulating CD4+ Tregs that have a number of anti-inflammatory functions [12, 15–17]. High levels of 1,25(OH)2D have been associated with anti-inflammatory lymphoid polarisation including a high proportion of Tregs [18]. Tregs induced or stimulated by vitamin D3 may play a role in controlling allo- and auto- immune T cell responses by releasing or expressing inhibitory cytokines such as IL-10 [19, 20] and TGF-beta [21], through release of granzymes and perforin [21] or via expression of inhibitory co-receptors such as CTLA-4 to prevent antigen presentation and initiation of the pro-inflammatory response [14]. There is therefore considerable animal or in vitro experimental evidence the vitamin D3 may indirectly or indirectly increase the numbers or function of Tregs.

There is also evidence from observational work or clinical trials in humans that higher vitamin D levels are associated with higher Treg/total T cell ratios and a more immunosuppressive phenotype [22, 23]. Several small studies with 30 to 60 participants have shown that supplementation with cholecalciferol administered orally with doses of VD3 that range from 12,000 to 30,000IU per week can increase the Treg/CD4 or Treg/CD3 ratio, absolute Treg concentrations in peripheral blood and the functional capacity of Tregs to supress effector cells in healthy individuals [23, 24] and in patients with inflammatory or autoimmune diseases [25–27]. Most of these studies defined Tregs as CD4+CD25+FOXP3+CD127lo. One study examined both thymic derived (CD31+) and peripherally induced Tregs (CCR7-) and found that both were enhanced, by 25% or more, after an intensive daily course of VD3 for 1 year in which a total of 850,000 IU were given [26].

Other vitamins and metabolites may affect T cell function. The function of Treg cells may also be increased by vitamin A supplements [28]. Moreover, the short chain fatty acid, butyrate, may be produced by commensal microbiota and prime Treg cells in the secondary lymphoid tissue of the gastrointestinal tract [29]. In mice, exogenous butyrate or the vitamin niacin or similar compounds acting via GPR109a, such as mono- or dimethyl-fumarate on lymphocytes, may increase Treg cell levels and function [30, 31]. Finally, gamma-amino butyric acid (GABA) has been used experimentally to increase Tregs in animals, but the effects of this butyrate derivative on Treg cell numbers and functions in humans is limited [32]. Altering the balance between pro-inflammatory phenotypes may be important in disease outcomes.

Effective, safe and inexpensive methods to increase Tregs would have wide application to reduce auto-immune, inflammatory diseases or to reduce complications of haematopoietic or solid organ transplantation. We have therefore undertaken a systematic review of randomised controlled trials (RCTs) to assess the efficacy of vitamin D, vitamin A, niacin and short-chain fatty acids and their derivatives in enhancing absolute Treg concentrations, Treg/Total T cell ratios, altered Treg phenotypes such as the ratio of naïve CD45RA+ Tregs to memory CD45RO+ Tregs, or Tregs expressing cutaneous or gut homing receptors (CLA1+ or alpha4beta7 integrin respectively) in patients with inflammatory or autoimmune disease.

## Methods

### Eligibility

This systematic review was conducted using a predefined protocol [33, 34] in accordance with Preferred Reporting Items for Systematic Reviews and Meta-Analyses (PRISMA) guidelines [35] (see Supplementary Information for PRISMA reporting checklist). Our eligibility criteria were as follows: (i) RCTs, (ii) patients with inflammatory or autoimmune disease or healthy participants; (iii) intervention (vitamin D or vitamin A or niacin or nicotinic acid or mono- or dimethyl-fumarate or Acipimox or butyrate or butyric acid or gamma-aminobutyric acid or gamma-aminobutyrate or 3-hydroxy-butyrate or 3-hydroxy-butyric acid, administered orally) compared with control or placebo; (iv) absolute concentration or proportion of Tregs measured. Studies which did not measure Tregs were excluded. Co-interventions were included only if administered to both/all trial arms. There were no other exclusion criteria.

### Search strategy and study selection

Studies were identified from searches to June 2019 of CENTRAL (*The Cochrane Library* 2018, Issue 4), MEDLINE (OvidSP, 1946 onwards), EMBASE (OvidSP, last 6 months), CINAHL (EBSCOHost, 1937 onwards), PUBMED (e-publications ahead of print only) and Web of Science (Conference Proceedings Citation Index—Science (CPCI-S), 1990 to present). Searches were not restricted by language or publication status. We also searched ongoing trial databases ClinicalTrials.gov and WHO International Clinical Trials Registry Platform (ICTRP). Full details of search strategies are provided in S1 Appendix. The titles and abstracts of all references were screened independently in duplicate by two reviewers against full eligibility criteria. Disagreements were resolved through discussion. The full text of papers was retrieved and screened for all those references for which a decision of eligibility could not be made from the title and abstract alone.

### Data extraction and analysis

Data were extracted onto customised data extraction forms by two reviewers independently; disagreements were resolved by consensus. Extracted data included study design, participant characteristics, interventions (dose, duration) and outcomes for each intervention group. Reviewers were not blinded to authorship, institutions, journals or the outcomes of trials. The risk of individual study bias was assessed using the Cochrane Collaboration’s tool for assessing risk of bias [36]. We assessed the following domains for each study: random sequence generation, allocation concealment, blinding of participants, personnel and outcome assessors, incomplete outcome data, selective reporting and other bias such as financial conflicts of interest. A three-point scale for bias was used–‘low’, ‘high’ and ‘unclear’ risk. The primary outcomes of this review were (i) absolute concentration of regulatory T-cells measured in peripheral blood, and (ii) proportion of Tregs measured in peripheral blood. Secondary outcomes were (i) Treg suppressive capacity (e.g. inhibition of expansion of effector T cells), (ii) correlation between serum vitamin D or vitamin A or niacin or short-chain fatty acid levels and the number or proportion of Tregs, (iii) safety measures (e.g. serum vitamin, serum calcium, C-reactive protein levels, excess infection or bleeding events), and (iv) compliance.

### Data analysis

Planned meta-analysis was not possible due to the paucity of studies and heterogeneous nature of participants, interventions and reported outcomes in the included studies. Analysis was therefore descriptive in nature, with conclusions drawn from tabulated results and comparisons across studies. Results were presented graphically in three studies [27, 37, 38]; values were estimated independently by two reviewers and the mean value taken. Where necessary, serum 25(OH)D levels were converted to ng/mL using the conversion 1 ng/mL = 2.5 nmol/L.

## Results

### Description of included studies

**Fig 1:**
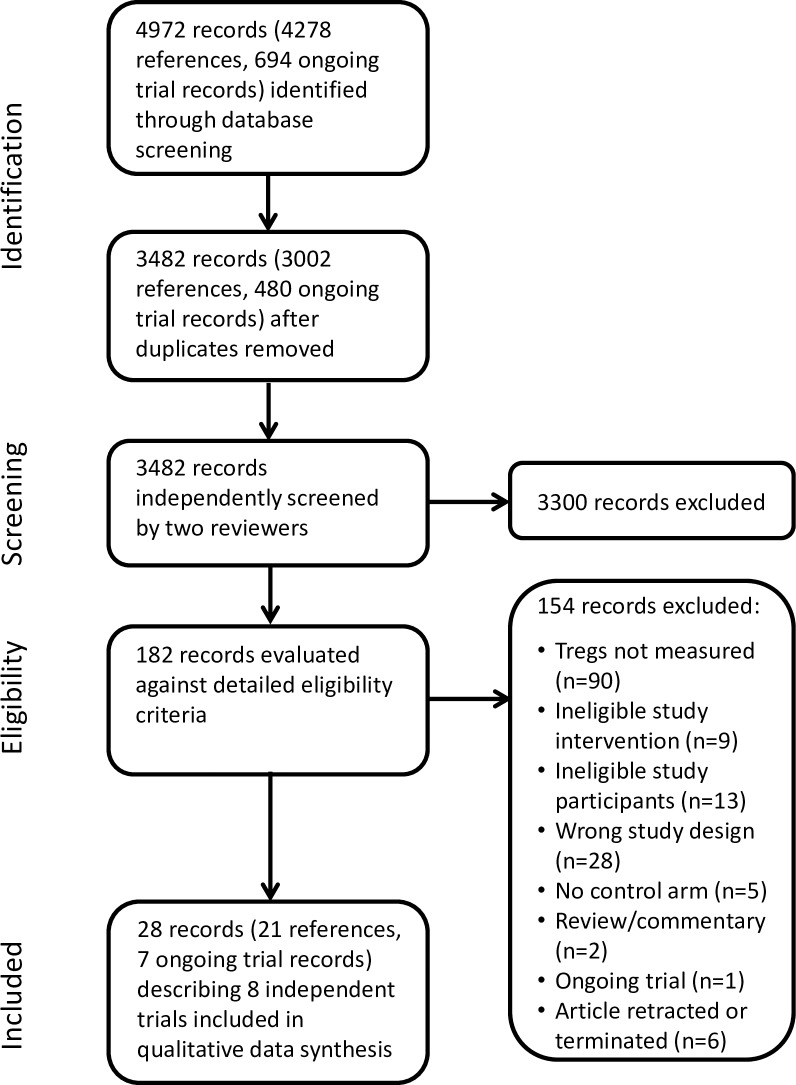
Study flow diagram showing the selection of eligible trials.

Searches of electronic databases and reference lists identified 4972 records (4278 references, 694 clinical trial records) (see Fig 1). From these, 11 full papers, ten conference abstracts and nine clinical trial records were identified which described a total of eight independent studies eligible for inclusion. One relevant ongoing trial was identified [39]. A summary of study participants and interventions is given in Table 1. Briefly, eligible studies comprised two trials of paediatric asthma [38, 40], three trials of type 1 diabetes [25, 27, 37], one trial of relapse-remitting multiple sclerosis [41], one trial of Addison’s disease [42] and one trial of healthy non-diabetic subjects described in two papers [23, 24], with a total of 322 randomised participants. All studies compared oral vitamin D to either placebo (7 studies) or control (1 study). The doses used in these studies were widely variable. Furthermore, all but two studies used doses of VD3 that were below those needed to increase systemic vitamin D levels to the upper limit of the normal range and below the maximum tolerated dose of 4000 IU/day. In these two trials, Muris and colleagues gave 7000 IU/day for first month, then 14,000 IU/day [41], whereas Penna-Martinez and colleagues gave 4000 IU/day for three months [42]. We did not identify any eligible studies of vitamin A, niacin or short-chain fatty acids and their derivatives which measured Tregs.

**Table 1:** Characteristics of included studies.

Condition	Type I Diabetes			Addison’s Disease	Multiple Sclerosis	Asthma		Healthy Controls
Study	Bogdanou 2017	Gabbay 2012	Treiber 2015	Penna-Martinez 2018	Muris 2016	Baris 2014	Majak 2009	Prietl 2014
**Participants**	Patients with recent onset of >2 months or chronic type 1 diabetes	Patients with a new diagnosis of type 1 diabetes (T1DM)	Young patients with new-onset type 1 diabetes	Women of childbearing age with Addison’s disease	Patients with relapse-remitting multiple sclerosis	Children aged 5–15 with mild/moderate persistent asthma (+/- rhinitis) sensitized to house dust mite	Children aged 6–12 years with IgE-dependent asthma assessed in outpatient setting	Healthy non-diabetic subjects
**Country of study**	Germany	Brazil	Austria	Germany	The Netherlands	Turkey	Poland	Austria
**Age**	Median 44 (IQR 33–52) years	Mean 18.6 (SD 2.7) years	Median 12.5 years	Median 48 (IQR 42–55) years	Mean 37.5 (SD 8.2) years	Mean 8.8 (SD 2.4) years	Range 6–12 years	Mean 33.5 (SD 10.5) years
**% Male**	51%	60%	68%	54%	42%	42%	61%	51%
**Randomised (intervention/ comparator)**	21/18 (analysed)	19/19	15/15	5/8	33/25	18/17	18/18	30/30
**Intervention**	Oral cholecalciferol (Vigantol) 4000 IU/day for three months (120,000 IU monthly)*	Oral cholecalciferol 2000 IU daily for 18 months (60,000 IU monthly)	Oral cholecalciferol (Oleovit D3) 70 IU/kg/day, weekly for 12 months (loading dose 140 IU/kg/day for 1 month) (4,200 IU for one month then 2,100 IU monthly)	Oral cholecalciferol (Vigantol) 4000 IU daily for 3 months**	Oral cholecalciferol (Vigantol) 7000 IU/day for first four weeks, then 14000 IU daily (420,000 IU monthly)	Oral vitamin D^#^ at 650 U daily for 12 months (19,500 IU monthly)	Oral cholecalciferol (Vigantoletten) 1000 IU/week for 3 months (4,000 IU monthly)	Oral cholecalciferol (Oleovit D3) 140,000 IU monthly for 3 months
**Comparator**	Placebo (details n/r)	Placebo (details n/r)	Placebo (peanut oil suspension)	Placebo (nr)	Placebo (details n/r)	Control (no placebo)	Placebo (wafer preparation with 0.3mg lactose)	Placebo (almond oil)
**Co-interventions**	None	Insulin (multiple [>3] daily injections with glucose self-monitoring	Insulin (dose n/r, adjustments of insulin dose made at each visit safety evaluation)	None	IFNbeta-1a, 44 μg three times weekly s.c. for a minimum of 90 days and not longer than 18 months	Subcutaneous immunotherapy; pharmacotherapy	Subcutaneous steroid immunotherapy for 12 months); corticosteroid (prednisone 20mg)	None
**Follow-up time points**	6, 12 weeks*	6, 12, 18 months	3, 6, 12 months	3 months**	48 weeks	6, 12 months	3, 12 months	4, 8, 12 weeks
**Measure of Tregs**	CD4^+^CD25^+^ CD127^dim/neg^ (absolute count)	% CD4^+^CD25^+^ Foxp3^+^ in peripheral blood	% CD4^+^CD25^hi^ FoxP3^+^ CD127^low^ in CD4^+^ cells	CD3^+^CD4^+^ CD25^bright^ CD127^dim/neg^ (absolute count)	% CD4^+^CD25^+^ CD127^neg^Foxp3^+^ in CD4^+^ cells***	% CD4^+^CD25^+^ Foxp3^+^ in peripheral blood	% CD4^+^CD25^+^ Foxp3^+^ in CD4^+^CD25^+^ cells	% CD4^+^CD25^hi^ FoxP3^+^ CD127^dim^ in CD4^+^ cells

A summary of individual study participants and interventions.

# Formulation of vitamin D was not specified.

*Followed by crossover to placebo/cholecalciferol at 12 weeks with follow-up at 18 and 24 weeks.

** Followed by crossover to placebo/cholecalciferol at 3 months with follow-up at 6 months.

*** also measured: CD4^+^CD25^+^CD127^neg^ and CD4^+^CD25^+^Foxp3^+^ in CD4^+^ cells.

n/r: not reported; s.c.: subcutaneously; SD: standard deviation.

### Quality assessment and risk of bias

**Fig 2:**
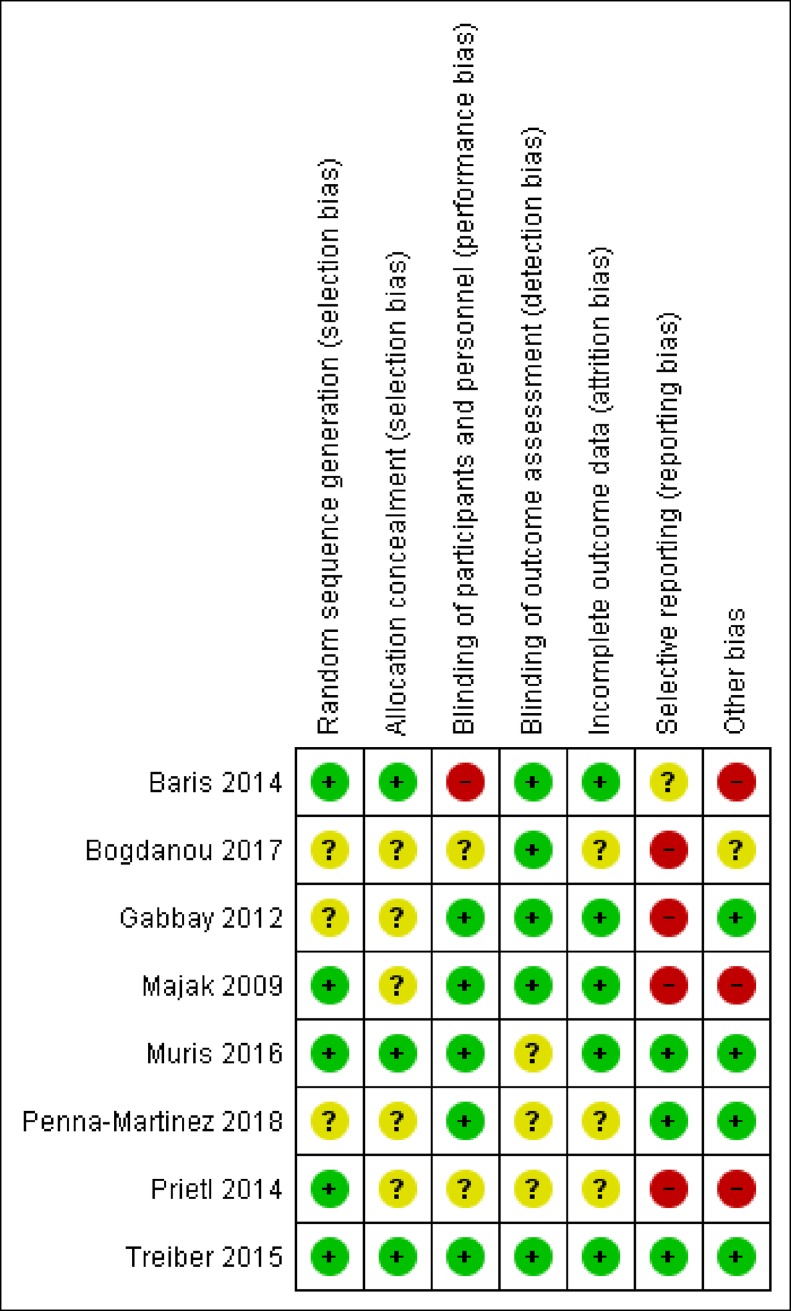
Risk of bias for each study assessed for systematic review. The domains of risk are shown for each study. Colour coding is used to depict a three-point scale for bias. Green (+) reflects a low score, red (-) a high score and yellow (?) an unclear risk of bias.

The risk of bias in individual trials is shown in Fig 2. Only one trial was low-risk in every domain [27]. One trial was subject to a risk of performance bias due to lack of blinding of participants [38]. A risk of reporting bias was identified in four trials, in which some outcomes defined in the methods [25] or trial protocol [37] were not reported, or differences in outcome definitions were identified between the trial protocol and the final study report [23, 40]; the risk of reporting bias was unclear in a fourth trial where no study protocol was identified. Two asthma trials and one multiple sclerosis trial administered immunotherapy as a co-intervention but with few details on the dose regimen or variation between trial arms, suggesting a potential high risk of bias [38, 40, 41]. Finally, a high risk of bias was attributed to differences in the inclusion criteria between the trial protocol and the final study report [23]. There are insufficient studies to conduct a formal test of publication bias as over ten articles are needed to yield meaningful results [43].

VD3 is synthesised in human epidermis by ultraviolet B conversion of 7-dehydrocholesterol (provitamin D3) and therefore sunlight directly influences the level of available VD3 [1, 2]. There may also be other seasonal effects that determine Treg numbers and function. However, none of the studies reported sun exposure or details of the timing of recruitment of subjects and controls, although the studies reported in this review did have control subjects given placebo. If these were contemporaneous controls, then they would largely control for differences in sunlight exposure, that may directly affect the formation of 25- hydroxyvitamin D3 and also seasonal effects. However, we cannot be sure studies have correctly recruited contemporaneous controls.

### Effect of interventions

#### Absolute concentration or proportion of regulatory T-cells

Two studies reported the absolute concentration of Tregs [37, 42]. In this small crossover trial of 39 type 1 diabetes patients [37], there was no difference between treatment arms in the intra-individual change in absolute concentration of Tregs (cells/ μl) (p > 0.2). In the latter crossover trial of Addison’s disease, there was similarly no difference between treatment arms (p = 0.35) [42].

Six studies reported the proportion of T regulatory cells (Tregs), defined as CD4^+^CD25^+^FoxP3^+^ measured in peripheral blood [25, 38] or as a proportion of CD4^+^CD25^+^ cells [40], CD4^+^CD25^hi^FoxP3^+^CD127^low^ as a proportion of CD4^+^ cells [23, 27], or CD4^+^CD25^+^CD127^neg^FoxP3^+^ as a proportion of CD4^+^ cells [41]. These measures do allow some qualitative comparison between trials.

In the five trials of immune-mediated disease patients, the results of these small trials do not show consistent changes in the proportion of Tregs after vitamin D supplementation. Baris and colleagues showed a significant difference in proportion of Tregs between groups at 12 months, but not at six months [38]. However, Gabbay and colleagues showed no significant differences between groups at any time point after supplementation [25]. Treiber and colleagues showed no significant differences between groups at any time point [27]; similarly, no significant differences between groups were found in the trial of multiple sclerosis [41]. In the fifth study, Majak and colleagues did not directly compare the difference in Treg proportions, only performing a three-way comparison with an additional placebo group which did not receive the co-intervention of steroids [40]. Furthermore, this study was confounded by the co-administration of oral steroids and vitamin D3.

**Fig 3:**
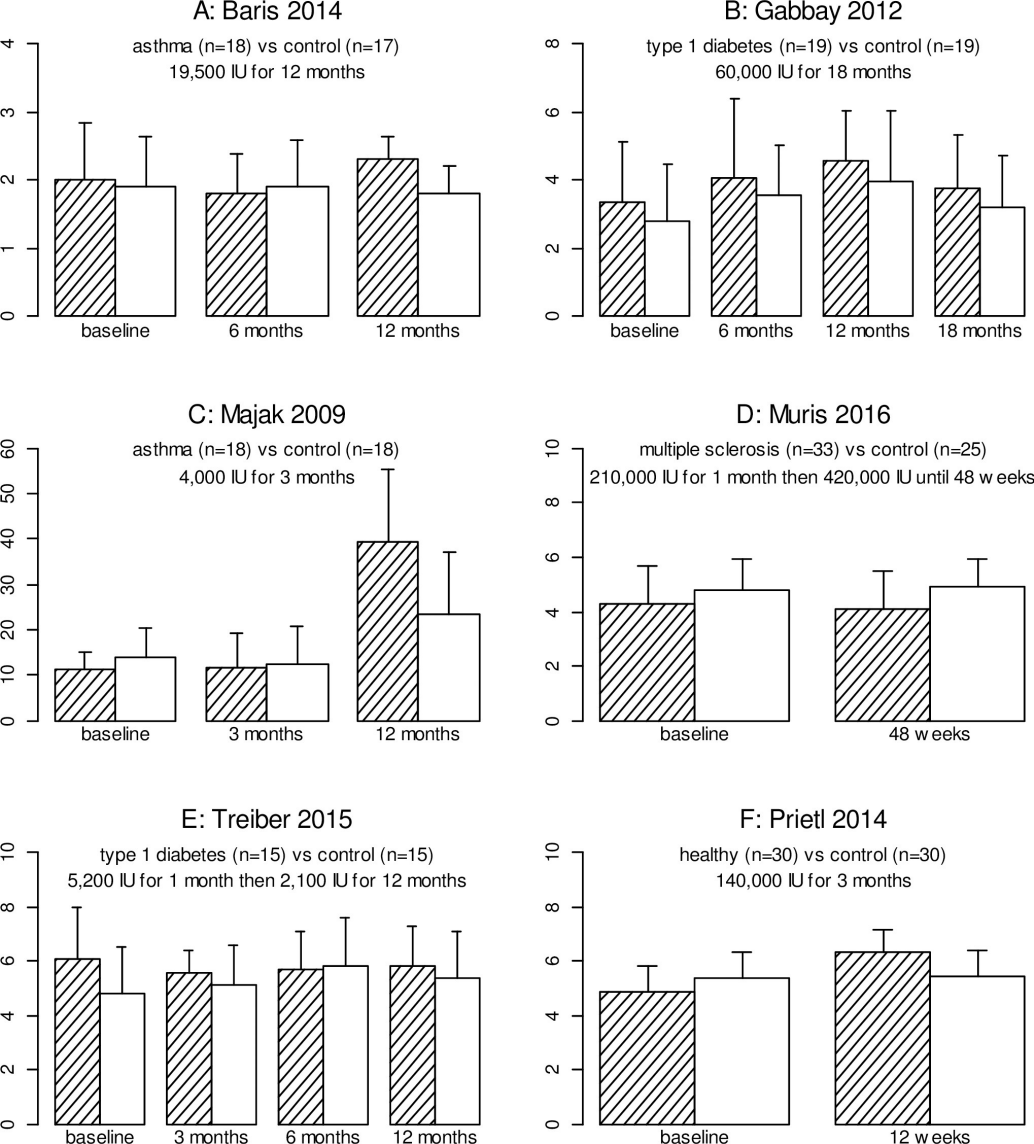
Mean % Tregs in subjects who received vitamin D (shaded bars) compared with those who received no vitamin D (unshaded bars), shown at timepoints measured in each study. Error bars show standard deviation. The percentage of Tregs is defined as (A, B) % CD4+CD25+ Foxp3+ in peripheral blood, (C) % CD4+CD25+ Foxp3+ in CD4+CD25+ cells, (D) CD4+CD25+ CD127negFoxp3+ in CD4+ cells, (E) % CD4+CD25hi FoxP3+ CD127low in CD4+ cells, (F) % CD4+CD25hi FoxP3+ CD127dim in CD4+ cells. The number randomised to each trial arm and the monthly equivalent doses of vitamin D in the treatment arm are shown.

However, comparison of results across studies (Fig 3) shows that the proportion of Tregs was higher in the vitamin D group compared to the control/placebo group at 12 months or longer in all but one trial, with a relative increase of Treg/CD4 or Treg/PBMCs ratios (using the respective definitions see above) associated with vitamin D supplementation ranging from 7.5% to 66.5% across these four studies. It is therefore plausible that these included trials were not sufficiently powered to find a significant effect on a change in the absolute concentration or proportion of regulatory T-cells.

In the trial of healthy subjects [23], a significant difference was reported between groups at 8 and 12 weeks with a higher percentage of Tregs observed in the VD3 group (at 12 weeks, mean 6.4% (SD 0.8%) (VD3) vs mean 5.5% (SD 1.0%) (placebo).

#### Treg suppressive capacity

Treg suppressive capacity was measured in two trials [23, 27], assessed as the reduction in % effector T cell proliferation in the presence of autologous Tregs.

Treiber and colleagues found a significantly higher suppressive capacity (reduction in Teffector proliferation in the presence of autologous Tregs) at 12 months (vitamin D: mean 37.2%, SD 25.0% vs placebo: mean 0.7%, SD 28.9% (p = 0.017)) [27]. Notably, the suppressive capacity at 12 months was significantly increased from baseline in the vitamin D group but significantly decreased in placebo group. These differences remained significant when corrected for baseline demographics (age, diabetes duration, BMI).

In healthy controls, Treg suppressive capacity was also increased in the vitamin D group (and decreased in placebo group) but these did not reach significance [23].

#### Correlation between serum vitamin D levels and measures of Tregs

One trial reported the correlation between serum 25(OH)D levels and Tregs in healthy controls [23]. Prietl and colleagues observed an increasing percentage of Tregs correlating significantly with increasing serum 25(OH)D from baseline to week 12 in the vitamin D group (Spearman’s rank order correlation of percentage Tregs and serum 25(OH)D (r = 0.339, p = 0.009).

One trial of type I diabetes measured the correlation between intra-individual changes of vitamin D /baseline vitamin D levels and intra-individual change in Tregs, and found no significant correlation (r = 0.11, p = 0.49) [37].

#### Safety

All eight trials measured serum 25(OH)D levels (Table 2). The baseline values of serum 25(OH)D were broadly similar with no significant differences found between treatment groups. After administration of vitamin D in the treatment arm, serum 25(OH)D levels were higher than in the control arm in all seven trials (Table 2). The mean (or median) concentration of serum 25(OH)D after vitamin D treatment was above the upper limit of normal range at 30 ng/mL but within acceptable upper safety limits of around 100 ng/mL [44, 45], even at the highest monthly equivalent dose of 420,000 IU [41]. The trials cannot define an optimum dose of vitamin D3 but they suggest that a better understanding of the dose-response relationship and optimising individual dosing may have the potential to increase therapeutic efficacy for vitamin D.

**Table 2:** Serum 25(OH)D levels.

Study	Vitamin D (Monthly equivalent dose)	Timepoint	Serum 25(OH)D (ng/mL) (mean, SD)	
			Vitamin D	Control
Baris 2014	19,500 IU for 12 months	Baseline	19 (9)	20 (12)
		12 months	31 (10)	20 (6)
Bogdanou 2017	120,000 IU for 3 months	Baseline	Median 19.5 (IQR 13.5–26) ^(a)^	Median 17 (IQR 9.5–23.5 ^(a)^
		3 months	Median 38 (IQR 34–41) ^(a)^	Median 16.5 (IQR 7.5–20) ^(a)^
Gabbay 2012	60,000 IU for 18 months	Baseline	26.3 (6.5)	25.8 (5.7)
		6 months	60.9 (21.6)	28.6 (8.8)
		18 months	65.0 (26.0)	40.5 (10.4)
Majak 2009	4,000 IU for 3 months	Baseline	32.0 (3.1)	31.3 (3.4)
		3 months	32.7 (2.5)	30.3 (2.9)
		12 months	n/r	n/r
Muris 2016	420,000 IU for 48 weeks ^(b)^	Baseline	Median 24.0 (IQR 15.2–34.0) ^(c)^	Median 21.6 (IQR 17.2–25.2) ^(c)^
		48 weeks	Median 92.4 (IQR 64.8–100.0) ^(c)^	Median 24.0 (IQR 14.4–34.0) ^(c)^
Penna-Martinez 2018	120,000 IU for 3 months	Baseline	19.4 (10.1) ^(a)^	17.9 (10.5) ^(a)^
		3 months	37.0 (11.4) ^(a)^	18.6 (9.8) ^(a)^
Prietl 2014	140,000 IU for 3 months	Baseline	25.5 (11.4)	25.8 (10.4)
		12 weeks	55.1 (18.1)	21.1 (9.8)
Treiber 2015	2,100 IU for 12 months ^(d)^	Baseline	24.0 (6.0) ^(^^a^^,^^c^^)^	30.0 (16.0) ^(^^a^^,^^c^^)^
		12 months	60.1 (24.0) ^(^^a^^,^^c^^)^	30.0 (16.0) ^(^^a^^,^^c^^)^

Serum 25(OH)D (ng/mL) values shown at baseline and end of trial in the intervention (vitamin D) and control/placebo (no Vitamin D) groups. n/r, not reported.

(a) values estimated from results reported graphically.

(b) 210,000 IU for first month.

(c) values converted from nmol/L.

(d) loading dose 4,200 IU for first month.

High levels of serum 25(OH)D may cause hypercalcaemia and monitoring serum concentrations of calcium is important to ensure the safety of study subjects. Serum (or plasma) calcium levels were measured in six trials [23, 25, 27, 37, 38, 42]. In vitamin D recipients, serum calcium levels remained within normal range throughout the study in all six trials. Two trials [23, 27] reported normal urinary calcium/creatinine ratio in both treatment arms. No excess infection or bleeding events were reported in any trial.

#### Compliance

Compliance with vitamin D treatment was assessed in three trials [25, 27, 37], although neither reported compliance rates. In one trial, two patients withdrew from the trial due to “insufficient adherence” to treatment [25] and in the second trial one patient was excluded after three months due to “intentional additional intake of vitamin D supplements” [27].

## Discussion

The potential for vitamin D to modulate the numbers and function of Tregs has been widely promoted after a considerable amount of data from ex vivo, in vitro or animal studies. The studies presented in this systematic review are consistent with an increase in the numbers and function of circulating Tregs after vitamin D supplementation. Five out of six trials (four in patients and one in healthy volunteers) showed an increase in circulating Tregs after periods of vitamin D supplementation from 3 to 12 months.

In one of the two trials where the function of Tregs was assessed there was an increase in Treg function after vitamin D supplementation [27] and in the other trial there was a trend for an increase in Treg function after vitamin D supplementation but this did not reach statistical significance [23]. It was not possible to combine the results from these two trials because of the different definitions and experimental conditions used to measure the capacity of Tregs to supress proliferation of T cells.

Moreover, this systematic review does not readily permit a formal meta-analysis of the difference in the number of Tregs as a proportion of CD4^+^ T cells between groups. The trials are not comparable because of the differences in dose of vitamin D, the differences in duration of treatment with vitamin D, and the different definition of Tregs, variously defined as the absolute number of CD4^+^CD25^+^CD127^dim/neg^ cells [37, 42], or expressed as a proportion: CD4^+^CD25^+^FoxP3^+^ cells [25, 38] or CD4^+^CD25^+^ cells [40], CD4^+^CD25^hi^FoxP3^+^CD127^low^ as a proportion of CD4^+^ cells [23, 27], or CD4^+^CD25^+^CD127^neg^FoxP3^+^ as a proportion of CD4^+^ cells [41].

It is clear that significant changes in vitamin D concentrations can be achieved through vitamin D supplementation. However, the nature of the dose-response relationship cannot be addressed by these data as all of the trials except one were conducted in patients with a range of pathologies, the doses of vitamin D varied from 420,000 IU/month to 4,000 IU/month, and the duration of dose ranged from 3 to 18 months. In any event, the crude average of vitamin D before the trials was 24.3 ng/ml and 24.5 ng/ml in experimental and control groups and after vitamin D supplementation was 57.0 ng/mL and 25.3 ng/mL in experimental and control groups. In the only trial conducted in healthy volunteers, the vitamin D concentration doubled after three months’ supplementation at 140,000 IU/month from 25.5 IU/mL to 55 IU/mL while declining from 25.8 IU/mL to 21.1 IU/mL in controls (seasonal variation in vitamin D concentrations is marked). In this trial, a higher Tregs/CD4 T cell ratio was observed in the vitamin D group (at 12 weeks, mean 6.4 (SD 0.8)% (vitamin D) vs mean 5.5 (SD 1.0) (placebo)), an increase of 16% (0.9/5.5 x 100%) [23].

Human trials designed to use vitamin D3 to reduce immune-mediated pathology in other clinical settings have been planned. O’Connell and colleagues are undertaking a randomised, placebo-controlled trial to study dose-related effects of vitamin D on immune responses in patients with a clinically defined syndrome of early multiple sclerosis and in healthy control participants, in which they will measure the frequency of CD4 T cell subsets [39]. We have shown that recipients of a haematopoietic stem cell graft with a higher proportion of Tregs to total CD3+ or CD4+ T cells have significantly improved clinical outcome [46, 47]. Since haematopoietic stem cell grafts are collected from the circulation after mobilisation of stem cells and lymphocytes from the donor’s bone marrow, it follows that enhancing Tregs in the circulation of the stem cell donor could reduce the risk of graft-versus-host disease in the recipient of the graft. Vitamin D3 could be also be used in recipients of haematopoietic stem cell grafts to enhance engraftment and reduce graft-versus-host disease. Preliminary results of a study to establish the pharmacokinetics of high-dose vitamin D3 in haematopoietic stem cell transplant recipients have been reported [48].

It is apparent that the recent clinical human studies in this review have demonstrated much less convincing changes in Treg numbers and functions than seen in animal models of human disease, where administration of 1α25 vitamin D3 successfully treats a range of autoimmune conditions, including antiretinal autoimmunity [49], acute colitis [50], diabetes [51], arthritis [52], experimental allergic encephalitis [53], asthma [54] and allogeneic haematopoietic stem cell transplantation [55]. These rodent models have shown a correlation between therapeutic efficacy and increased frequency or quantity of CD4+CD25+ T cells and expression of molecules associated with down regulation of anti-inflammatory responses including IL-10, TGF-β, and CTLA-4. This may be due to insufficient doses and/or duration of treatment, genetic or environmental factors in human populations or differences in measurement of Treg numbers and location.

One further factor may be the preparation of vitamin D used. The human studies used cholecalciferol or vitamin D3, but biological activity requires formation of the mono-hydroxylated 25(OH) vitamin D3 in liver by vitamin D 25-hydroxylase [56] and then hydroxylation at the 1-α position by 25-hydroxyvitamin D3 1-alpha-hydroxylase to form calcitriol 1,25-dihydroxycholecalciferol or 1,25(OH)2D which is the ligand for the vitamin D receptor [57]. Many murine studies used the di-hydroxylated 1, 25 vitamin D [49, 51, 53, 54] and expression and activity of the vitamin D 25-hydroxylase may be variable in expression or activity between subjects, or be systematically down regulated after vitamin D3 supplementation.

The clinical studies of vitamin D3 in volunteers are limited and the single randomised trial by Prietl and colleagues did report an increase in Treg/CD4 ratios after three months of 140,000 IU per months [23]. Higher doses of vitamin D may be possible as adverse effects have been reported only at 25(OH)D serum concentrations above 150 nmol/L (60 ng/mL) and an intake of ≥10,000 IU (250 μg) per day [5].

It is likely that the response of Tregs to vitamin D3 varies between individuals and there is some evidence that effects of increasing vitamin D3 on Treg numbers might be more effective in vitamin D deficient individuals [58]. This is important as such differential response may have substantially reduced the power of smaller studies to show a true difference between groups given vitamin D3 and placebo and none of the included trials performed such a sub-group analysis. It would be very helpful for future trials of vitamin D3 as an immunomodulatory agent to include estimates of the baseline vitamin D3 levels in the trial design, power calculations and sub-group analysis. Further studies to examine the effect of vitamin D on Treg numbers and function must be adequately powered to access clinically significant changes in numbers and function over shorter (1–3 months) and longer term (6–12 months). The doses of vitamin D3 given are below the maximum tolerated dose and optimising the dose of vitamin D for short- and long-term supplementation may help to clarify the effect of the vitamin on clinically significant immunological parameters. The effect of supplementation on sub-sets of Tregs has not been explored. There is very limited data on the effect of co-administration of oral steroids and vitamin D3 on Tregs and given the wide-spread use of oral steroids in many auto-immune diseases, this may be a useful area for future research.

The review has several limitations. Our search strategy for this systematic review was restricted to those trials which measured Tregs. We acknowledge that this approach may lead to a degree of publication bias arising not only from unpublished studies, but from selective reporting of results in studies which found no effect of the intervention on Tregs. Unfortunately, there are insufficient studies to conduct a formal test of publication bias. We did not review other outcomes of immunomodulation such as cytokine production that could be considered in future, separate reviews of the role of vitamin D and other small molecules on immunomodulation. Moreover, the studies identified for the review have many limitations being small, relatively underpowered and with one study identified examining the effect of vitamin D on normal volunteers. None of the trials gave adequate information regarding the timing of recruitment of controls or sun exposure which have significant effects on vitamin D3 and Treg numbers and function. As described above, no further meta-analysis of the results of individual studies was possible because of heterogeneity in the study population, vitamin D dose and duration and definition and methods for measuring Treg numbers and function. It would be helpful to have some standard definitions of efficacy for such trials.

It is possible blood samples may not be the best tissue for analyses of the effects of vitamin D on Treg cell numbers or function. It is quite likely that the number and function of Tregs differs from tissue to tissue. However, this review can only examine published data and no trial reported on the concentration or function of Tregs in tissues.

The review has shown evidence from one trial of normal volunteers that vitamin D3 supplementation may significantly increase Treg/CD4 ratios, and that vitamin D3 supplementation may increase Treg function, as demonstrated in a separate trial of type I diabetes patients. However, other small trials in patients with auto-immune disease are underpowered to show clinically significant changes in Tregs as a proportion of CD4 cells. There is clearly potential for further well designed and suitably powered clinical studies aimed at improving the numbers of T regulatory cells for clinical benefit.

## Supporting information

S1 Appendix Search strategy. (DOCX)

S2 Appendix PRISMA checklist. (DOC)
