# Supplementary material for: The role of vitamin D in increasing circulating T regulatory cell numbers and modulating T regulatory cell phenotypes in patients with inflammatory disease or in healthy volunteers: A systematic review
Source: PLoS One. 2019 Sep 24;14(9):e0222313. doi: 10.1371/journal.pone.0222313 (PMC6759203; doi:10.1371/journal.pone.0222313)
Supplement: S1 Appendix — (DOCX) [file pone.0222313.s001.docx]

**S1 Appendix: Search Strategy**

The following databases were searched for RCTs and systematic reviews to 25 June 2019:

CENTRAL (*The Cochrane Library* 2019, issue 6)

MEDLINE (OvidSP, 1946 onwards)
Embase (OvidSP, 1974 onwards)

CINAHL (EBSCOHost, 1937 onwards)

PubMed (epublications ahead of print only)

Web of Science (Conference Proceedings Citation Index- Science (CPCI-S) -1990 to present)

Ongoing Studies:

ClinicalTrials.gov

WHO International Clinical Trials Registry Platform (ICTRP)

**Search Strategies:**

**CENTRAL (*The Cochrane Library*)**

#1 MeSH descriptor: [Vitamin D] explode all trees

#2 MeSH descriptor: [Vitamin A] explode all trees

#3 MeSH descriptor: [Niacin] this term only

#4 MeSH descriptor: [beta Carotene] this term only

#5 MeSH descriptor: [Niacinamide] explode all trees

#6 (vitamin D* or dihydroxyvitamin* or hydroxycholecalciferol* or dihydroxycholecalciferol* or hydroxycalciferol* or hydroxyergocalciferol* or hydroxyvitamin* or calciferol* or doxercalciferol or ergocalciferol* or ercalcidiol or calcifediol or calcidiol or calcifediol or calderol or calcijex or cholecalciferol* or colecalciferol* or dedrogyl or hectorol or hidroferol or calcitriol* or bocatriol or calcijex or decostriol or osteotriol or paricalcitol or renatriol or rocaltrol or silkis or sitriol or soltriol or tirocal or calcamine or dihydrotachysterin* or dihydrotachysterol* or tachystin or tocopherol* or vigantol or zemplar*)

#7 (trans-retinol or aquasol A or retinol or vitamin A* or retinoic acid* or trans-retinoic acid* or tretinoin or retin A or retinyl palmitate or vesanoid or beta carotene or multivitamin* or multi-vitamin*)

#8 (niacin* or nicotinic acid or pyridinecarboxylic acid orenduracin or induracin or nicotinate or nicamin or "nico 400" or nico400 or nicobid or nicocap or wampocap or pyridinecarboxamide or "vitamin B3" or enduramide or nicotinsaureamid or nicobion or nicotinamide or papulex or "vitamin pp" or "vitamin p-p")

#9 MeSH descriptor: [Fumarates] explode all trees

#10 (dimethyl fumarate or dimethylfumarate or tecfidera or BG-12 or monoethyl fumarate or monoethylfumarate or fumaric acid or fumaderm or acipimox or olbetam)

#11 MeSH descriptor: [Butyrates] explode all trees

#12 (short chain fatty acid* or SCFA* or butyr* or but?noic acid or butanoate or BodyBio or tributyrin or tributyrate or phenylbutyrate or hydroxybutyr*)

#13 #1 or #2 or #3 or #4 or #5 or #6 or #7 or #8 or #9 or #10 or #11 or #12

#14 MeSH descriptor: [T-Lymphocytes] explode all trees

#15 MeSH descriptor: [Forkhead Transcription Factors] this term only

#16 ("T regulatory lymphocyte" or "T regulatory lymphocytes" or "T regulatory cell*" or "T regulatory cells" or "T cell" or "T cells" or Tcell* or "T lymphocyte" or "T lymphocytes" or "immunoregulatory cell" or "immunoregulatory cells" or "regulatory cell" or "regulatory cells" or "T reg" or "T regs" or Treg* or nTreg* or iTreg* or FoxP3* or "fox p3" or "forkhead box P3" or "Foxp 3" or scurfin or lymphocytes or (cell* near/2 marker*) or CD25* or CD4* or CD3* or CD8* or CD27* or "peripheral blood")

#17 #14 or #15 or #16

#18 #13 and #17

#19 MeSH descriptor: [Dietary Supplements] this term only

#20 (supplement* or tablet* or capsule* or pill or pills or lozenge* or oral* or daily or drug* or bolus or administ* or dose* or dosage or dosing or slow releas* or extended releas* or treatment* or therap*)

#21 #19 or #20

#22 #18 and #21

#23 MeSH descriptor: [Vitamin D] explode all trees and with qualifier(s): [AD, TU]

#24 MeSH descriptor: [Vitamin A] explode all trees and with qualifier(s): [AD, TU]

#25 MeSH descriptor: [beta Carotene] this term only and with qualifier(s): [AD, TU]

#26 MeSH descriptor: [Butyrates] explode all trees and with qualifier(s): [AD, TU]

#27 MeSH descriptor: [Niacin] this term only and with qualifier(s): [AD, TU]

#28 MeSH descriptor: [Fumarates] explode all trees and with qualifier(s): [AD, TU]

#29 MeSH descriptor: [Niacinamide] this term only and with qualifier(s): [AD, TU]

#30 MeSH descriptor: [Vitamins] explode all trees and with qualifier(s): [AD, TU]

#31 #23 or #24 or #25 or #26 or #27 or #28 or #29 or #30

#32 #17 and #31

#33 #22 or #32

**MEDLINE (OvidSP)**

1. exp Vitamin D/

2. exp Vitamin A/ or Beta Carotene/

3. Niacin/ or Niacinamide/

4. (vitamin D* or dihydroxyvitamin* or hydroxycholecalciferol* or dihydroxycholecalciferol* or hydroxycalciferol* or hydroxyergocalciferol* or hydroxyvitamin* or calciferol* or doxercalciferol or ergocalciferol* or ercalcidiol or calcifediol or calcidiol or calcifediol or calderol or calcijex or cholecalciferol* or colecalciferol* or dedrogyl or hectorol or hidroferol or calcitriol or bocatriol or calcijex or calcitriolnefro or decostriol or osteotriol or paricalcitol or renatriol or rocaltrol or silkis or sitriol or soltriol or tirocal or calcamine or dihydrotachysterin* or dihydrotachysterol* or tachystin or abortosan or tocopherol* or vigantol or zemplar*).tw,kf.

5. (retinyl palmitate or trans-retinol or aquasol A or retinol or vitamin A* or retinoic acid* or trans-retinoic acid* or tretinoin or retin A or vesanoid or beta carotene or multivitamin* or multi-vitamin*).tw,kf.

6. (niacin* or nicotinic acid or pyridinecarboxylic acid orenduracin or induracin or nicotinate or nicamin or "nico 400" or nico400 or nicobid or nicocap or wampocap or pyridinecarboxamide or "vitamin B3" or enduramide or nicotinsaureamid or nicobion or nicotinamide or papulex or "vitamin pp" or "vitamin p-p").tw,kf.

7. exp Butyrates/

8. (short chain fatty acid* or SCFA* or butyr* or but?noic acid or butanoate or BodyBio or tributyrin or tributyrate or phenylbutyrate or hydroxybutyr*).tw,kf.

9. exp Fumarates/

10. (dimethyl fumarate or dimethylfumarate or tecfidera or BG-12 or monoethyl fumarate or monoethylfumarate or fumaric acid or acipimox or olbetam).tw,kf.

11. 1 or 2 or 3 or 4 or 5 or 6 or 7 or 8 or 9 or 10

12. T-Lymphocytes/ or exp CD4-positive T-lymphocytes/ or exp CD8-positive T-lymphocytes/

13. Forkhead Transcription Factors/

14. (T regulatory lymphocyte* or T regulatory cell* or T cell* or Tcell* or T lymphocyte* or immunoregulatory cell* or regulatory cell* or T reg* or Treg* or nTreg* or iTreg* or FoxP3* or fox p3* or forkhead box P3 or "Foxp 3" or scurfin or lymphocytes or (cell* adj2 marker*) or CD25* or CD4* or CD3* or CD8* or CD27* or peripheral blood).tw,kf.

15. 12 or 13 or 14

16. 11 and 15

17. Dietary Supplements/

18. (supplement* or tablet* or capsule* or pill or pills or lozenge* or oral* or daily or drug* or bolus or administ* or dose* or dosage or dosing or slow releas* or extended releas* or treatment* or therap*).tw,kf.

19. 17 or 18

20. 16 and 19

21. Vitamins/ad, tu or exp Vitamin D/ad, tu or exp Vitamin A/ad, tu or Niacin/ad, tu or Niacinamide/ad, tu or Beta Carotene/ad, tu or exp Butyrates/ad, tu or exp Fumarates/ad, tu

22. 15 and 21

23. 20 or 22

24. Meta-Analysis.pt.

25. ((meta analy* or metaanaly*) and (trials or studies)).ab.

26. (meta analy* or metaanaly* or evidence-based).ti.

27. ((systematic* or evidence-based) adj2 (review* or overview*)).tw.

28. (cochrane or medline or pubmed or embase or cinahl or cinhal or lilacs or citation index or psyclit or psychlit or psycinfo or psychinfo or "web of science" or scopus).ab.

29. Cochrane Database of systematic reviews.jn.

30. ((literature or systematic* or comprehensive* or electronic*) adj2 search*).ab.

31. (additional adj (papers or articles or sources)).ab.

32. (bibliograph* or handsearch* or hand search* or manual* search* or searched or reference list*).ab.

33. (relevant adj (journals or articles)).ab.

34. or/24-33

35. Review.pt.

36. RANDOMIZED CONTROLLED TRIALS AS TOPIC/

37. selection criteria.ab. or critical appraisal.ti.

38. (data adj (extraction or analys$)).ab.

39. RANDOMIZED CONTROLLED TRIALS/

40. or/36-39

41. 35 and 40

42. 34 or 41

43. randomized controlled trial.pt.

44. controlled clinical trial.pt.

45. randomi*.tw.

46. placebo.ab.

47. clinical trials as topic.sh.

48. randomly.ab.

49. groups.ab.

50. trial.tw.

51. or/43-50

52. 42 or 51

53. (ANIMALS/ or exp ANIMAL EXPERIMENTATION/ or exp MODELS, ANIMAL/) not HUMANS/

54. Editorial.pt.

55. 53 or 54

56. 52 not 55

57. 23 and 56

**EMBASE (OvidSP)**

1. exp Vitamin D/

2. exp Retinoid/

3. Beta Carotene/

4. Nicotinamide/ or Nicotinamide Derivative/

5. Nicotinic Acid/

6. (vitamin D* or dihydroxyvitamin* or hydroxycholecalciferol* or dihydroxycholecalciferol* or hydroxycalciferol* or hydroxyergocalciferol* or hydroxyvitamin* or calciferol* or doxercalciferol or ergocalciferol* or ercalcidiol or calcifediol or calcidiol or calcifediol or calderol or calcijex or cholecalciferol* or colecalciferol* or dedrogyl or hectorol or hidroferol or calcitriol or bocatriol or calcijex or calcitriolnefro or decostriol or osteotriol or paricalcitol or renatriol or rocaltrol or silkis or sitriol or soltriol or tirocal or calcamine or dihydrotachysterin* or dihydrotachysterol* or tachystin or vigantol or zemplar*).tw.

7. (retinyl palmitate or trans-retinol or aquasol A or retinol or vitamin A* or retinoic acid* or trans-retinoic acid* or tretinoin or retin A or vesanoid or beta carotene or multivitamin* or multi-vitamin*).tw.

8. (niacin* or nicotinic acid or pyridinecarboxylic acid orenduracin or induracin or nicotinate or nicamin or "nico 400" or nico400 or nicobid or nicocap or wampocap or pyridinecarboxamide or "vitamin B3" or enduramide or nicotinsaureamid or nicobion or nicotinamide or papulex or "vitamin pp" or "vitamin p-p").tw.

9. Short Chain Fatty Acid/ or Butyric Acid/ or Butyric Acid Derivative/

10. (short chain fatty acid* or SCFA* or butyr* or but?noic acid or butanoate or BodyBio or tributyrin or tributyrate or phenylbutyrate or hydroxybutyr*).tw.

11. Fumaric Acid Derivative/

12. (dimethyl fumarate or dimethylfumarate or tecfidera or BG-12 or monoethyl fumarate or monoethylfumarate or fumaric acid or acipimox or olbetam).tw.

13. 1 or 2 or 3 or 4 or 5 or 6 or 7 or 8 or 9 or 10 or 11 or 12

14. exp T Lymphocyte/

15. Forkhead Transcription Factor/

16. (T regulatory lymphocyte* or T regulatory cell* or T cell* or Tcell* or T lymphocyte* or immunoregulatory cell* or regulatory cell* or T reg* or Treg* or nTreg* or iTreg* or FoxP3* or fox p3* or forkhead box P3 or "Foxp 3" or scurfin or lymphocytes or (cell* adj2 marker*) or CD25* or CD4* or CD3* or CD8* or CD27* or peripheral blood).tw.

17. 14 or 15 or 16

18. 13 and 17

19. Diet Supplementation/

20. Vitamin Supplementation/

21. (supplement* or tablet* or capsule* or pill or pills or lozenge* or oral* or daily or drug* or bolus or administ* or dose* or dosage or dosing or slow releas* or extended releas* or treatment* or therap*).tw.

22. 19 or 20 or 21

23. 18 and 22

24. Meta Analysis/

25. (meta analy* or metaanaly* or evidence-based).ti.

26. ((meta analy* or metaanaly*) and (trials or studies)).ab.

27. Systematic Review/

28. ((systematic* or evidence-based) adj2 (review* or overview*)).tw.

29. (evidence synthes* or cochrane or medline or pubmed or embase or cinahl or cinhal or lilacs or "web of science" or science citation index or scopus or search terms or literature search or electronic search* or comprehensive search* or systematic search* or published articles or search strateg* or reference list* or bibliograph* or handsearch* or hand search* or manual* search*).ab.

30. ((electronic* or online) adj (sources or resources or databases)).ab.

31. ((additional adj (papers or articles or sources)) or (relevant adj (journals or articles))).ab.

32. or/24-31

33. Review.pt.

34. (data extraction or selection criteria).ab.

35. 33 and 34

36. 32 or 35

37. Editorial.pt.

38. 36 not 37

39. crossover-procedure/ or double-blind procedure/ or randomized controlled trial/ or single-blind procedure/

40. (random* or factorial* or crossover* or cross over* or cross-over* or placebo* or doubl* blind* or singl* blind* or assign* or allocat* or volunteer*).mp.

41. 39 or 40

42. 38 or 41

43. limit 42 to (conference abstracts or embase)

44. 23 and 43

45. limit 44 to dd=20160704-20170717

**CINAHL (EBSCOHost)**

S1 (MH "Vitamin D+") OR (MH "Vitamin A") OR (MH "Beta Carotene")

S2 TI ( (vitamin D* OR dihydroxyvitamin D* OR hydroxycholecalciferol* OR dihydroxycholecalciferol* OR hydroxycalciferol* OR hydroxyergocalciferol* OR hydroxyvitamin D* OR calciferol* OR doxercalciferol OR ergocalciferol* OR ercalcidiol OR calcifediol OR calcidiol OR calcifediol OR calderol OR calcijex OR cholecalciferol* OR colecalciferol* OR dedrogyl OR hectorol OR hidroferol OR dihydroxyvitamin* D* OR calcitriol* OR bocatriol OR calcijex OR decostriol OR osteotriol OR paricalcitol OR renatriol OR rocaltrol OR silkis OR sitriol OR soltriol OR tirocal OR calcamine OR dihydrotachysterin OR dihydrotachysterol OR tachystin OR ercalcidiol OR zemplar) ) OR AB ( (vitamin D* OR dihydroxyvitamin D* OR hydroxycholecalciferol* OR dihydroxycholecalciferol* OR hydroxycalciferol* OR hydroxyergocalciferol* OR hydroxyvitamin D* OR calciferol* OR doxercalciferol OR ergocalciferol* OR ercalcidiol OR calcifediol OR calcidiol OR calcifediol OR calderol OR calcijex OR cholecalciferol* OR dedrogyl OR hectorol OR hidroferol OR dihydroxyvitamin* D* OR calcitriol OR bocatriol OR calcijex OR calcitriolnefro OR decostriol OR osteotriol OR paricalcitol OR renatriol OR rocaltrol OR silkis OR sitriol OR soltriol OR tirocal OR calcamine OR dihydrotachysterin OR dihydrotachysterol OR tachystin OR vigantol OR ercalcidiol OR zemplar) )

S3 TI ( (trans-retinol OR aquasol A OR retinol OR vitamin A* OR retinoic acid* OR trans-retinoic acid* OR tretinoin OR retin A OR vesanoid OR multivitamin* OR multi-vitamin*) ) OR AB ( (trans-retinol OR aquasol A OR retinol OR vitamin A* OR retinoic acid* OR trans-retinoic acid* OR tretinoin OR retin A OR vesanoid OR multivitamin* OR multi-vitamin*) )

S4 (MH "Niacin")

S5 TI ( (niacin* or nicotinic acid or pyridinecarboxylic acid orenduracin or induracin or nicotinate or nicamin or "nico 400" or nico400 or nicobid or nicocap or wampocap or pyridinecarboxamide or "vitamin B3" or enduramide or nicotinsaureamid or nicobion or nicotinamide or papulex or "vitamin pp" or "vitamin p-p") ) OR AB ( (niacin* or nicotinic acid or pyridinecarboxylic acid orenduracin or induracin or nicotinate or nicamin or "nico 400" or nico400 or nicobid or nicocap or wampocap or pyridinecarboxamide or "vitamin B3" or enduramide or nicotinsaureamid or nicobion or nicotinamide or papulex or "vitamin pp" or "vitamin p-p") )

S6 (MH "Butyric Acids+")

S7 TI ( (short chain fatty acid* or SCFA* or butyr* or but?noic acid or butanoate or BodyBio or tributyrin or tributyrate or phenylbutyrate or hydroxybutyr* OR dimethyl fumarate or dimethylfumarate or tecfidera or BG-12 or monoethyl fumarate or monoethylfumarate or fumaric acid or fumaderm or acipimox or olbetam) ) OR AB ( (short chain fatty acid* or SCFA* or butyr* or but?noic acid or butanoate or BodyBio or tributyrin or tributyrate or phenylbutyrate or hydroxybutyr* OR dimethyl fumarate or dimethylfumarate or tecfidera or BG-12 or monoethyl fumarate or monoethylfumarate or fumaric acid or fumaderm or acipimox or olbetam) )

S8 S1 OR S2 OR S3 OR S4 OR S5 OR S6 OR S7

S9 (MH "T Lymphocytes")

S10 TI ( (T regulatory lymphocyte* or T regulatory cell* or T cell* or Tcell* or T lymphocyte* or immunoregulatory cell* or regulatory cell* or T reg* or Treg* or nTreg* or iTreg* or FoxP3* or fox p3* or forkhead box P3 or "Foxp 3" or scurfin or lymphocytes or (cell* adj2 marker*) or CD25* or CD4* or CD3* or CD8* or CD27* or peripheral blood) ) OR AB ( (T regulatory lymphocyte* or T regulatory cell* or T cell* or Tcell* or T lymphocyte* or immunoregulatory cell* or regulatory cell* or T reg* or Treg* or nTreg* or iTreg* or FoxP3* or fox p3* or forkhead box P3 or "Foxp 3" or scurfin or lymphocytes or (cell* adj2 marker*) or CD25* or CD4* or CD3* or CD8* or CD27* or peripheral blood) )

S11 S9 OR S10

S12 S8 AND S11

S13 TX (supplement* or tablet* or capsule* or pill or pills or lozenge* or oral* or daily or drug* or bolus or administ* or dose* or dosage or dosing or slow releas* or extended releas* or treatment* or therap*)

S14 S12 AND S13

**PubMed**

#1 ("vitamin D" OR "vitamin D2" OR "vitamin D3" OR dihydroxyvitamin* OR hydroxycholecalciferol* OR dihydroxycholecalciferol* OR hydroxycalciferol* OR hydroxyergocalciferol* OR "hydroxyvitamin D" OR "hydroxyvitamin D2" OR "hydroxyvitamin D3" OR calciferol* OR doxercalciferol OR ergocalciferol* OR ercalcidiol OR calcifediol OR calcidiol OR calcifediol OR calderol OR calcijex OR cholecalciferol* OR colecalciferol* OR dedrogyl OR hectorol OR hidroferol OR calcitriol* OR bocatriol OR calcijex OR decostriol OR osteotriol OR paricalcitol OR renatriol OR rocaltrol OR silkis OR sitriol OR soltriol OR tirocal OR calcamine OR dihydrotachysterin OR dihydrotachysterol OR tachystin OR tocopherol* OR zemplar* OR ercalcidiol OR retinyl palmitate OR trans-retinol OR "aquasol A" OR retinol OR "vitamin A" OR retinoic acid* OR trans-retinoic acid* OR tretinoin OR "retin A" OR vesanoid OR multivitamin* OR multi-vitamin* OR niacin* OR "nicotinic acid" OR pyridinecarboxylic acid OR enduracin OR induracin OR nicotinate OR nicamin OR "nico 400" OR nico400 OR nicobid OR nicocap OR wampocap OR pyridinecarboxamide OR "vitamin B3" OR enduramide OR nicotinsaureamid OR nicobion OR nicotinamide OR papulex OR "vitamin pp" OR "vitamin p-p" OR short chain fatty acid* OR SCFA* OR butyr* OR butanoic acid OR butenoic acid OR butanoate OR BodyBio OR tributyrin OR tributyrate OR phenylbutyrate OR hydroxybutyr* OR dimethyl fumarate OR dimethylfumarate OR tecfidera OR BG-12 OR monoethyl fumarate OR monoethylfumarate OR fumaric acid OR fumaderm OR acipimox OR olbetam OR vigantol)

#2 ("T regulatory lymphocyte" OR "T regulatory lymphocytes" OR "T regulatory cell*" OR "T regulatory cells" OR "T cell" OR "T cells" OR Tcell* OR "T lymphocyte" OR "T lymphocytes" OR "immunoregulatory cell" OR "immunoregulatory cells" OR "regulatory cell" OR "regulatory cells" OR "T reg" OR "T regs" OR Treg* OR nTreg* OR iTreg* OR FoxP3* OR "fox p3" OR "forkhead box P3" OR "Foxp 3" OR scurfin OR lymphocytes OR "marker cell" OR "marker cells" OR CD25* OR CD4 OR CD3 OR CD8* OR CD27* OR "peripheral blood")

#3 (supplement* OR tablet* OR capsule* OR pill OR pills OR lozenge* OR oral* OR daily OR drug* OR bolus OR administ* OR dose* OR dosage OR dosing OR slow releas* OR extended releas* OR treatment* OR therapy OR therapies OR therapeutic*)

#4 (random* OR blind* OR "control group" OR placebo* OR controlled OR groups OR trial* OR "systematic review" OR "meta-analysis" OR metaanalysis OR "literature search" OR medline OR pubmed OR cochrane OR embase OR LILACS) AND (publisher[sb] OR inprocess[sb] OR pubmednotmedline[sb])

#5 #1 AND #2 AND #3 AND #4

**WEB OF SCIENCE CPCS-C**

#1 TS=("vitamin D" OR "vitamin D2" OR "vitamin D3" OR dihydroxyvitamin* OR hydroxycholecalciferol* OR dihydroxycholecalciferol* OR hydroxycalciferol* OR hydroxyergocalciferol* OR "hydroxyvitamin D" OR "hydroxyvitamin D2" OR "hydroxyvitamin D3" OR calciferol* OR colecalciferol* OR doxercalciferol OR ergocalciferol* OR ercalcidiol OR calcifediol OR calcidiol OR calcifediol OR calderol OR calcijex OR cholecalciferol* OR dedrogyl OR hectorol OR hidroferol OR calcitriol* OR bocatriol OR calcijex OR decostriol OR osteotriol OR paricalcitol OR renatriol OR rocaltrol OR silkis OR sitriol OR soltriol OR tirocal OR calcamine OR dihydrotachysterin OR dihydrotachysterol OR tachystin OR tocopherol* OR zemplar* OR ercalcidiol OR retinyl palmitate OR trans-retinol OR "aquasol A" OR retinol OR "vitamin A" OR retinoic acid* OR trans-retinoic acid* OR tretinoin OR "retin A" OR vesanoid OR multivitamin* OR multi-vitamin* niacin* OR "nicotinic acid" OR pyridinecarboxylic acid orenduracin OR induracin OR nicotinate OR nicamin OR "nico 400" OR nico400 OR nicobid OR nicocap OR wampocap OR pyridinecarboxamide OR "vitamin B3" OR enduramide OR nicotinsaureamid OR nicobion OR nicotinamide OR papulex OR "vitamin pp" OR "vitamin p-p" OR short chain fatty acid* OR SCFA* OR butyr* OR butanoic acid OR butenoic acid OR butanoate OR BodyBio OR tributyrin OR tributyrate OR phenylbutyrate OR hydroxybutyr* OR dimethyl fumarate OR dimethylfumarate OR tecfidera OR BG-12 OR monoethyl fumarate OR monoethylfumarate OR fumaric acid OR fumaderm OR acipimox OR olbetam OR vigantol)

#2 TS=("T regulatory lymphocyte" OR "T regulatory lymphocytes" OR "T regulatory cell*" OR "T regulatory cells" OR "T cell" OR "T cells" OR Tcell* OR "T lymphocyte" OR "T lymphocytes" OR "immunoregulatory cell" OR "immunoregulatory cells" OR "regulatory cell" OR "regulatory cells" OR "T reg" OR "T regs" OR Treg* OR nTreg* OR iTreg* OR FoxP3* OR "fox p3" OR "forkhead box P3" OR "Foxp 3" OR scurfin OR lymphocytes OR "marker cell" OR "marker cells" OR CD25* OR CD4 OR CD3 OR CD8* OR CD27* OR "peripheral blood")

#3 TS=(supplement* OR tablet* OR capsule* OR pill OR pills OR lozenge* OR oral* OR daily OR drug* OR bolus OR administ* OR dose* OR dosage OR dosing OR "slow release" OR "slow releasing" OR "extended release" OR "extended releasing" OR treatment* OR therapy OR therapies OR therapeutic*)

#4 TS=(random* OR blind* OR "control group" OR placebo* OR controlled OR groups OR trial* OR "systematic review" OR "meta-analysis" OR metaanalysis OR "literature search" OR medline OR pubmed OR cochrane OR embase OR LILACS)

#5 #1 AND #2 AND #3 AND #4

**ClinicalTrials.gov (Expert Search)**

Search Terms: "T regs" OR Tregs OR "T regulatory" OR "T cells" OR "regulatory cells" OR immunoregulatory
Intervention: vitamin OR cholecalciferol OR colecalciferol OR retinol OR retanoic OR multivitamin OR niacin OR "short chain fatty acid" OR "short chain fatty acids" OR butanoic OR butyrate OR fumaric OR fumarate OR acipimox OR olbetam OR vigantol
OR
( "T regs" OR Tregs OR "T regulatory" OR "T cells" OR "regulatory cells" OR immunoregulatory ) AND EXACT "Interventional" [STUDY-TYPES] AND ( randomly OR randomised OR randomized ) AND ( vitamin OR cholecalciferol OR colecalciferol OR retinol OR retanoic OR multivitamin OR niacin OR "short chain fatty acid" OR "short chain fatty acids" OR butanoic OR butyrate OR fumaric OR fumarate OR acipimox OR olbetam OR vigantol ) [TREATMENT]

**ICTRP**

Title: (T regs OR Tregs OR T regulatory OR T cells OR regulatory cells OR immun*)

Interventions: (vitamin OR cholecalciferol OR colecalciferol OR retinol OR retanoic OR multivitamin OR niacin OR short chain fatty acid OR butanoic OR butyrate OR fumaric OR fumarate OR acipimox OR olbetam OR vigantol)
